# Distal spinal nerve development and divergence of avian groups

**DOI:** 10.1038/s41598-020-63264-5

**Published:** 2020-04-14

**Authors:** Dana J. Rashid, Roger Bradley, Alida M. Bailleul, Kevin Surya, Holly N. Woodward, Ping Wu, Yun-Hsin (Becky) Wu, Douglas B. Menke, Sergio G. Minchey, Ben Parrott, Samantha L. Bock, Christa Merzdorf, Emma Narotzky, Nathan Burke, John R. Horner, Susan C. Chapman

**Affiliations:** 1 Department of Cell Biology and Neuroscience, Montana State University, Bozeman, MT 59717 USA; 2 Department of Microbiology and Immunology, Montana State University, Bozeman, MT 59717 USA; 3 Key Laboratory of Vertebrate Evolution and Human Origins, Institute of Vertebrate Paleontology and Paleoanthropology, Chinese Academy of Sciences, Beijing 100044, China and CAS Center for Excellence in Life and Paleoenvironment, 100044 Beijing, China; 4 Honors College, Montana State University, Bozeman, MT 59717 USA; 5 Department of Anatomy and Cell Biology, Oklahoma State University Center for Health Sciences, Tulsa, OK 74107 USA; 6 Keck School of Medicine, University of Southern California, Los Angeles, CA 90033 USA; 7 Department of Genetics, University of Georgia, Athens, GA 30602 USA; 8 Savanah River Ecology Laboratory and Odum School of Ecology, University of Georgia, Athens, GA 30602 USA; 9 American Studies Program, Montana State University, Bozeman, MT 59717 USA; 10 Department of Biological Sciences, Clemson University, Clemson, SC 29634 USA; 11 Honors Program, Chapman University, Orange, CA 92866 USA

**Keywords:** Developmental biology, Evolution, Neuroscience

## Abstract

The avian transition from long to short, distally fused tails during the Mesozoic ushered in the Pygostylian group, which includes modern birds. The avian tail embodies a bipartite anatomy, with the proximal separate caudal vertebrae region, and the distal pygostyle, formed by vertebral fusion. This study investigates developmental features of the two tail domains in different bird groups, and analyzes them in reference to evolutionary origins. We first defined the early developmental boundary between the two tail halves in the chicken, then followed major developmental structures from early embryo to post-hatching stages. Differences between regions were observed in sclerotome anterior/posterior polarity and peripheral nervous system development, and these were consistent in other neognathous birds. However, in the paleognathous emu, the neognathous pattern was not observed, such that spinal nerve development extends through the pygostyle region. Disparities between the neognaths and paleognaths studied were also reflected in the morphology of their pygostyles. The ancestral long-tailed spinal nerve configuration was hypothesized from brown anole and alligator, which unexpectedly more resembles the neognathous birds. This study shows that tail anatomy is not universal in avians, and suggests several possible scenarios regarding bird evolution, including an independent paleognathous long-tailed ancestor.

## Introduction

Vertebrate tails are highly divergent in form and function. In birds, tails have evolved adaptations specific for flight and sexual selection. Extant bird tails are composed of the proximal region, characterized by unfused (‘free’) caudal vertebrae, and the distal region harboring the pygostyle, a bony structure formed from fusion of the distal caudals. The observation that pygostyle fusion occurs progressively after hatching^1^ led us to question how the unique avian tail morphology is rooted in early developmental events, and whether these events are consistent across all three major groups of extant birds (neoaves, galloanseriforms, and paleognaths).

The modern bird tail originated in the Mesozoic era, at the long- to short-tailed transition^2^. *Archaeopteryx*, the earliest known bird, sported a long, reptilian-like tail with greater than 20 caudal vertebrae^3,4^. While *Archaeopteryx* fossil specimens were discovered in Germany, the greatest cache of Mesozoic bird fossils has been found in the Jehol beds in China, representing a period between 131 to 120 million years ago^5^. Jehol specimens indicate that both long and short-tailed birds coexisted at this critical time in avian evolution. The short-tailed birds exhibited a number of unique morphologies, among them the occurrence of the pygostyle and additional bone fusions throughout the axial and peripheral skeleton^6^. With the exception of *Rahonavis*, a specimen from Madagascar that may or may not have been avian^7^, in the last 120 million years, only short-tailed birds have been documented. No adult avian species with intermediate morphologies, such as short tails lacking a pygostyle, have been identified.

Mutational analyses have demonstrated that proper development of certain structures is critical to formation of a full length and/or unfused tail^8^. These structures include (but are not limited to) somites, intervertebral discs, intersomitic blood vessels, notochord, neural tube, cartilage, and neural crest; their breadth underscores the wide variety of genetic modifications that can result in a truncated tail and/or fused caudal vertebrae. A relevant example is the genetic change underlying the truncated tail phenotype in the rumpless araucana chicken. In these birds, tissue that normally contributes to axial extension instead differentiates to a neural fate, resulting in multiple neural tubes, complete loss or reduction of tail vertebrae, and irregular caudal vertebrae fusion^9^. The observed ectopic neural tissue indicates that investigation of the development of individual tail structures may shed light on evolutionary changes in bird tail morphology.

The development of many distal tail structures is not well described, which complicates correlations with adult morphologies and investigations into relatedness between different bird groups. This, in turn, hinders evolutionary analyses of avian lineages. In this study, the boundary between the pygostyle and free vertebrae was defined in the chicken, and the major tail structures were followed from early embryo to hatching. Placing our findings in the context of evolution, the differences observed between the two tail regions were also evaluated in representatives of the three major modern bird groups, as well as in American alligator and brown anole. These studies expose variation in distal neural development that indicates tail-specific evolutionary events in neognathous and paleognathous birds.

## Results and Discussion

To investigate caudal development in birds and how that development correlates with long-tailed reptiles, we pursued a methodology whereby we first defined the avian pygostyle and free vertebrae boundary in the chicken. Pre-hatching ontogeny analysis of particular structures within the tail was subsequently conducted in representatives of the three major groups of birds, including chicken and quail for galloanseriforms, rock dove for neoaves, and emu for paleognaths. The avian species examined have similar caudal vertebrae counts; nine in the chicken and quail (5 free vertebrae and 4 pygostyle vertebrae; our observations), nine to ten in the rock dove (5 to 6 free vertebrae and 4 pygostyle vertebrae^10^ and our observations), and eight in the emu (5 free vertebrae and three pygostyle vertebrae (our observations from MOR 186 and three post-hatch emu carcasses). Pre-hatching developmental analysis was then performed in alligator and brown anole (both have approximately 40 caudal vertebrae^11,12^), to compare to avian. Because our investigation was necessarily limited to only a few species, and we were also interested in manifestation of adult morphologies from earlier developmental events, we expanded our analyses to include adult avian tails, including adult avian museum specimens and microCT scans of two kiwi specimens.

### Establishing the embryonic boundary between pygostyle and free vertebral elements in the chicken

**Figure 1:**
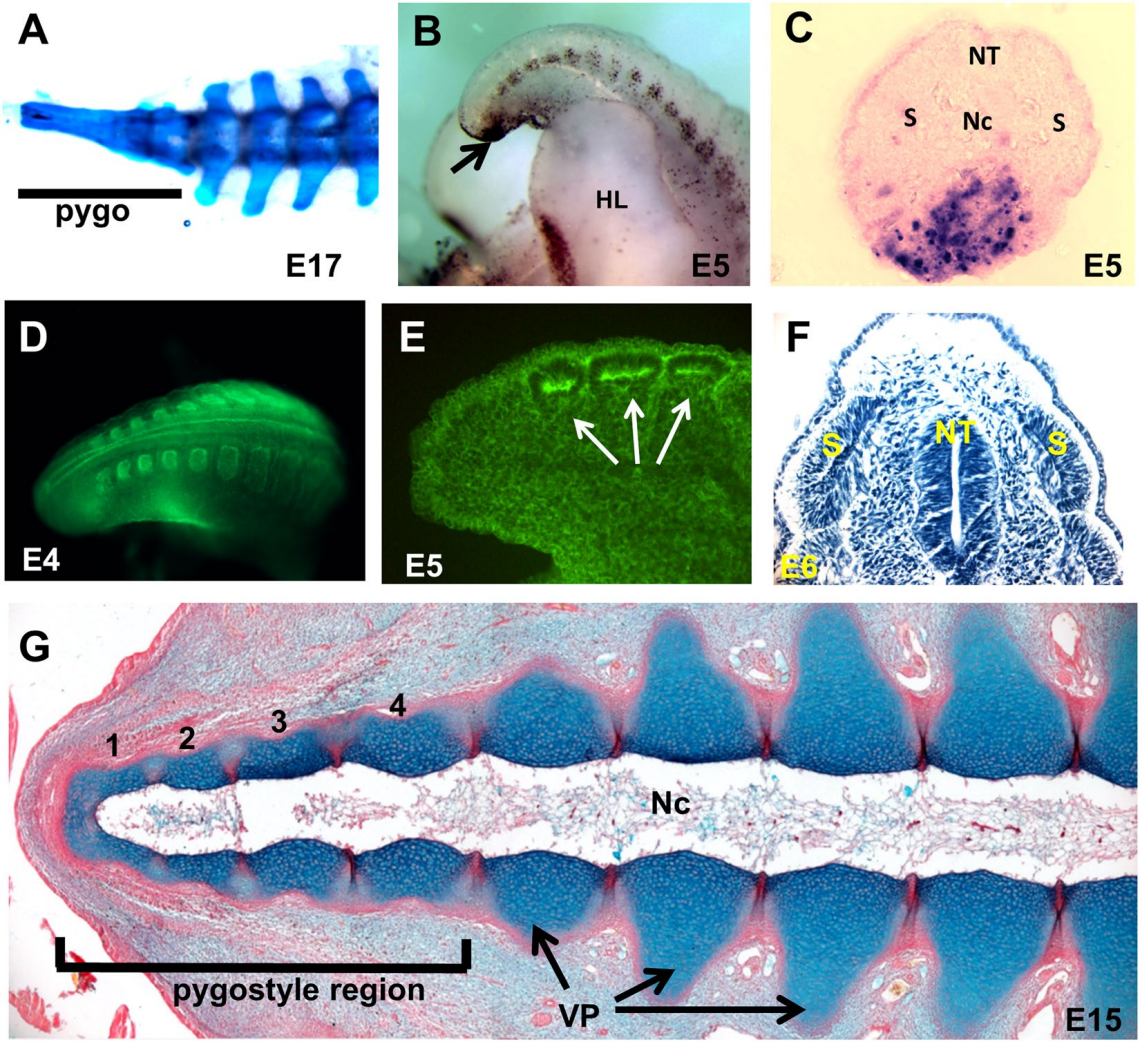
Defining the pygostyle to free vertebrae boundary in the chicken. (**A**) E17 Alcian blue and alizarin red stained wholemount tail. At E17, ossification has not yet commenced, but transverse processes differences are readily observed between pygostyle pre-vertebrae and free caudal vertebrae. (**B**) TUNEL-stained E5 wholemount, indicating ventral, distal apoptosis in the chick embryo tail (arrow). The segmented pattern of apoptosis in the tail is in ventral lateral dermamyotome. (**C**) Cross-section of a TUNEL-stained chick embryo tail (E5), showing apoptosis is ventral and excluded from somite domains. (**D,E**) Phalloidin 488 stained somites in chick embryo tails. Wholemount E4, D and E, E5 cryosection. (**F**) Hematoxylin-stained oblique coronal cryosection, E6, showing discreet distalmost somites. (**G**) E11 coronal paraffin section, Alcian blue and picrosirius red stained, on the same plane as the notochord. The pygostyle region encompasses the distalmost four pre-vertebrae, and these elements lack vertebral processes. Abbreviations: HL: hindlimb; Nc: notochord; NT: neural tube; pygo: pygostyle; S, somite; VP, vertebral process.

In order to evaluate development between the pygostyle and free vertebrae regions, we first needed to establish the boundary between them. The precise number of somite elements that contribute to the bony pygostyle is not known in any bird species. Considering the wide spectrum of avian pygostyle shapes and sizes, this number likely varies^1,13–15^. Since fusion or loss of vertebral elements could theoretically occur at multiple developmental stages, and thereby affect the inclusive number of pygostyle elements, progressive stages were examined for morphology and cell death in chicken embryos. The stages that were evaluated include somite formation to subsequent chondrogenesis. When the vertebral cartilage models are established, the chicken pygostyle-specific prevertebrae can be distinguished from free caudal prevertebrae by their lack of lateral processes (Fig. 1A) (for definitions of terms, and more information specific to those terms, see Glossary of Terms, Supplementary File).

TUNEL assays performed at E4 through E7 stages confirm that apoptosis does not affect terminal somites, in agreement with other studies^16–20^. Robust cell death is only observed in the remnant of the tailbud mesenchyme (especially at E4 to E5), and in the ventrolateral dermomyotome (Fig. 1B,C). That cell death occurs primarily outside of somite domains supports the notion that resorption of somites does not occur.

Another factor that could affect the number of contributing somites to the pygostyle is whether somites fully segregate from each other, and from presomitic mesoderm. During proper development, somites form as discrete units. However, segregation anomalies, where somites fail to establish proper borders, can ultimately lead to fully or partially fused vertebrae^8,21,22^, and thus could affect the number of pygostyle specific elements. To test whether somite segregation failure occurs in the pygostyle region, we examined distal somite morphology in the chick embryo. Chick embryo tails from E4 to E6 (HH22-29) were stained with phalloidin (Fig. 1D,E), or with hematoxylin (Fig. 1F). Phalloidin stains actin filaments, and clearly delineates somites that have undergone a mesenchymal to epithelial transition and have separated from the paraxial mesoderm. The phalloidin and hematoxylin data show that progressively smaller somites form towards the tail tip, and they all form discretely with no evidence of segregation anomalies, including segregation of the most distal somite from posterior mesoderm. This data complements several other studies that show that when somite addition has ceased, undifferentiated mesenchyme remains at the tail tip, distal from the last separate somite, which then undergoes apoptosis^9,23,24^. Our study and those previously reported demonstrate that even the most distal somite segregates, and that all distal somites form discretely. Combined with the lack of apoptosis, the number of somites therefore remains constant in chicken tail development.

A constant number of somites allows for uncomplicated tracing of pygostyle elements throughout development from the somite forming stage onwards. At E7, staining with Alcian blue alone results in what appears to be a fully fused, single element pygostyle (E17, Fig. 1A). However, the Alcian blue and picrosirius red double stain shows unequivocally that the pre-vertebral elements are distinct and separated by intervertebral discs (E15, Fig. 1G). By following progressive stages, we observed that the distal four somites contribute to the distal four cartilaginous prevertebrae that lack transverse processes. These progressively fuse in the distal to proximal direction into the pygostyle structure in the first four months after hatching^1^. The free caudal vertebrae arise from the next proximal five somites, amounting to nine total vertebral elements in the chicken tail. While some variability is observed between individuals and between different chicken breeds, these numbers were consistent within the inbred white leghorn, Cornish rock, and bovan brown breeds we examined. Defining the number of pygostyle elements sets the boundary between the pygostyle and free vertebrae regions, which is critical in determining whether distinct early developmental processes specific to pygostyle formation can be identified.

### Development of structures in the pygostyle and free vertebrae regions. Intersomitic blood vessels, notochord, neural tube

**Figure 2:**
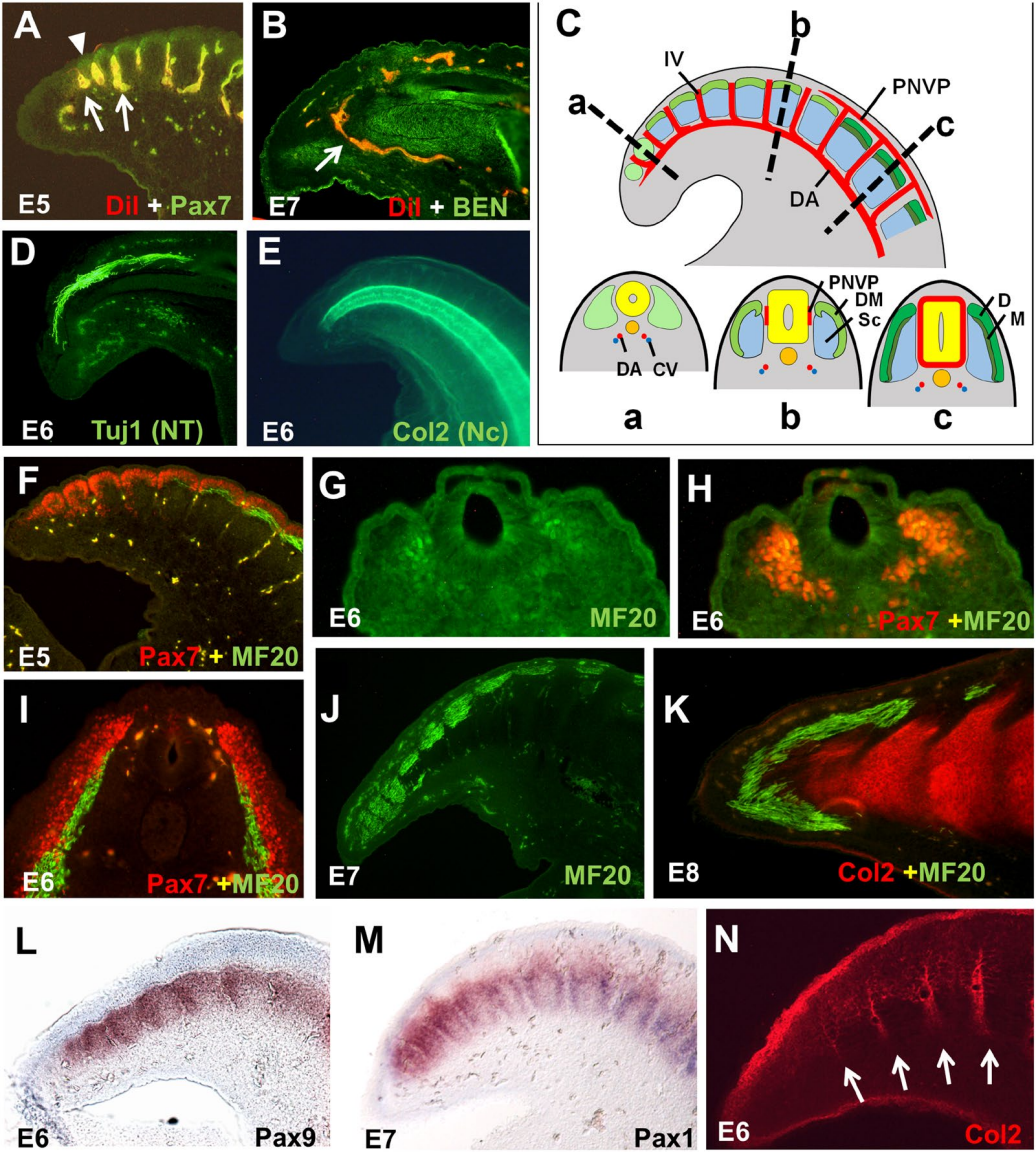
Development of major tail structures in the chicken. (**A**,**B**). Vasculature, stained by DiI microinjection. (**A**) E5 sagittal cryosection, co-stained for vasculature (DiI, red) and somites (Pax7, green). DiI bleeds into both the 488λ and 568λ channels with our imaging system, and therefore fluoresces as yellow or orange in the merged images. Intersomitic blood vessels (indicated by arrows) form between all somites, including the most distal (indicated by arrowhead). (**B**) E7 sagittal cryosection, stained for vasculature (DiI) and neural tissue (BEN, green). The dorsal aorta (arrow) connects to the vascular network surrounding the neural tube at the distal end of the tail. (**C**) Diagram of E5 tail, showing somite maturation and axial vasculature. Somite addition has completed by E5. Upper, sagittal view; lower, cross-sections as indicated in sagittal view. At E5, somites in the tail exhibit a full range of maturation, from newly formed, undifferentiated somites at the distal end to matured somites segregated into dermatome, myotome and sclerotome at the proximal end (neural tube in yellow; notochord in orange). (**D**) E6 sagittal cryosection stained with Tuj1 mAb, which recognizes neurons within the neural tube (NT). (**E**) E6 wholemount tail stained with Col2 mAb, which recognizes the notochord (Nc). Neural tube and notochord taper distally and both terminate just shy of the tail tip. (**F**) E5 tail, sagittal cryosection, co-stained for somitic mesoderm and dermamyotome/dermatome (Pax7, red) and myotome (MF20, green). (**G,H**) Cross-section of E6 tail tip stained for myotome (MF20, **G**), and myotome plus dermatome (MF20 + Pax7, **H**). Expression is partially overlapping at this time and place, indicating somite compartments have not yet fully differentiated. (**I**) Cross-section of E6 tail, in the free vertebrae region, stained for myotome (MF20) and dermatome (Pax7). (**J**) E7 tail, sagittal cryosection, stained for myotome (MF20), which has extended to the distal tail and differentiated from other somite compartments by this stage. (**K**) E8 sagittal cryosection, stained for prevertebrae (Col2) and myotome (MF20). By E8, the myotome has formed a cap around the distalmost prevertebra. (**L**) Pax9 ISH, sagittal cryosection, showing robust expression in the distal tail at E6. (**M**) Pax1 ISH, sagittal cryosection, also showing robust expression at E7. (**N**) Von Ebner’s fissures (arrows) stained by Col2 at stage E6, indicating early development of intervertebral discs in the pygostyle region. Abbreviations: CV: caudal vein; D: dermatome; DA: dorsal aorta; DM: dermamyotome; IV: intersomitic blood vessel; M: myotome; PNVP: perineural vascular plexus; Sc: sclerotome.

We examined several processes known to be involved in tail shortening and/or caudal vertebrae fusion^8^. We hypothesized that one or more of these processes would differ between the pygostyle and free vertebrae regions, which would hint at the evolutionary change(s) that gave rise to the modern avian tail. One such candidate is the intersomitic blood vessels that form upon the addition of each somite. We microinjected DiI into the vitelline arteries in ovo from E5 to E7, and observed intersomitic vasculature between all somites throughout the tail, including all pygostyle-specific somites (Fig. 2A). By E7, the dorsal aorta blood vessels wrap dorsally around the most distal somite, and connect to the perineural vascular network (PNVN) that surrounds the neural tube (Fig. 2B). The intersomitic blood vessels also connect into the PNVN^10,25^. The vasculature at the end of the tail is illustrated in Fig. 2C. The development of intersomitic blood vessels between even the most distal somites indicates that this process is not impeded in the pygostyle region, and supports the finding that all tail somites are formed separately.

Examination of the chicken neural tube (Fig. 2D) and notochord (Fig. 2E) also supported analogous development in the pygostyle and free caudal vertebrae domains. Immunostaining showed that both taper distally, in proportion to the tail profile, and extend to the tail tip. While excess neural differentiation and defects in notochord patterning can cause short and/or fused tails^8,9^, there is no morphological evidence of such events in wildtype chicken development.

### Somite maturation and intervertebral discs

In the mouse, the most prevalent mutations that cause tail truncation and/or caudal vertebrae fusion affect somites^8^. These mutations span the breadth of somite development, affecting segregation, differentiation, polarity, resegmentation, or condensation prior to chondrogenesis. Somite development, therefore, was a prime candidate to examine as a potential causative agent in the avian short fused tail.

Somite differentiation was examined in pygostyle and free vertebrae regions. Somite markers included Pax7, which is expressed in early whole somites but is restricted to the dermomyotome (and then dermatome) as somites mature^26,27^. MF20, an antibody specific for sarcomeric myosin, was used as a marker for myotome^28^. At E5, the tail exhibits a wide range of somite maturation, with nascent, undifferentiated somites at the tail tip and somites compartmentalized into dermatome, myotome and sclerotome at the proximal end of the tail (Fig. 2C,F). At E6, dermomyotome has subdivided to dermatome and myotome, and myotome is present at the tail tip (Fig. 2G). Although myotome is not clearly separated from the dermatome at E6 (Fig. 2H) as it is more proximally in the tail (Fig. 2I), its complete segregation in the distal tail is observed at E7 (Fig. 2J). By E8, the myotome has formed a cap around the distal end of the axial column (Fig. 2K), presumably to initiate musculature formation in the uropygium (a complex comprised of the pygostyle, rectricial bulbs, rectrices, and associated skin, connective tissues, and musculature). Dermatome of the distal somites was not examined beyond the somite stage, but likely follows the precedent of more proximal regions, and contributes to the skin, in this case of the uropygium.

Pax1 and Pax9 contribute to vertebral chondrogenesis and intervertebral disc formation^29,30^, and an earlier study^31^ suggested that their decreased protein expression in the chick embryo synsacrum could be linked to the fusion of synsacral vertebrae. To test whether *pax1* and *pax*9 gene expression is decreased in the pygostyle sclerotomes relative to the free vertebrae region, we analyzed their expression patterns by *in situ* hybridization. Sclerotome staining of these genes was equivalent in both tail domains (Fig. 2L,M), indicating no reduction of expression. The differences between our tail data and the Peters *et al*. synsacrum findings suggest the need for future studies investigating the potential alternate strategies of vertebral fusion in the pygostyle and the synsacrum. Relevant to this study, no significant differences were observed in compartmentalization and the fates of somite compartments between the pygostyle and free vertebrae tail regions.

**Figure 3:**
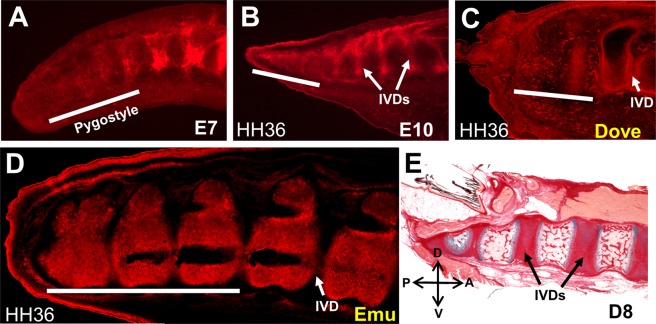
Sclerotome polarity differences between chicken, dove, and emu. (**A,B**), chicken. (**A**) Peanut agglutinin (PNA) staining at E7, showing poorly defined sclerotome polarity in the pygostyle region. (**B**) PNA staining at E10 (HH36). At this stage, PNA staining is diffuse in the pygostyle region, but preferentially stains intervertebral disc domains in the free vertebrae region of the tail. (**C**) Rock dove, PNA staining at HH36 stage. Like chicken, the rock dove (a neoave) exhibits poorly defined PNA staining in the pygostyle region. (**D**) Emu, PNA staining at HH36 stage. Unlike chicken and rock dove, emu PNA staining at this stage stains sclerotome and neural arches, with no perceptible staining differences between the pygostyle and free vertebrae tail regions. Also unlike chicken and rock dove, intervertebral discs are unstained. White bars indicate extent of pygostyle region. (**E**) Chicken pygostyle region, 8 days post-hatching (D8), sagittal view of distal tail, Alcian blue/picrosirius red staining. Intervertebral discs are fully formed between all pygostyle-specific vertebrae. Abbreviations: A: anterior, D: dorsal, IVD: intervertebral disc, P: posterior, V: ventral.

After compartmentalization, the sclerotome resegments^32,33^. Resegmentation in the chicken pygostyle region results in the formation of intervertebral discs, and is further supported by detection of von Ebner’s fissures, the features that give rise to the discs^34,35^. Von Ebner’s fissures were stained with collagen II antibody (Fig. 2N), and intervertebral discs were stained with picrosirius red in cartilage stages (Figs. 1G; 3E; also^1^). Perturbations in chicken somite resegmentation in the pygostyle region were not detected.

While proper somite anterior/posterior (AP) polarity is required for resegmentation^21,36^, resegmentation results in sclerotomes with renewed AP polarity. We questioned whether this subsequent polarity is fully established in the chicken pygostyle region. Perturbations in chicken dorsoventral somite polarity have been observed in distal somites^37^, but AP polarity had not yet been addressed. AP sclerotome polarity between E5 and E10 was investigated using peanut agglutinin (PNA) lectin staining, which labels first the posterior halves of sclerotomes after resegmentation^38^, and in later stages preferentially stains intervertebral disc regions in chick embryos (our observations). The PNA target influences the migration of neural crest cells, specifically to inhibit migration into the posterior halves of sclerotomes^38,39^. We find that PNA staining in the chicken E5 to E8 embryo is nearly absent in the pygostyle region, unlike in the free vertebrae tail region (Fig. 3A). At E10 (HH36), PNA in the pygostyle domain diffusely stains the pre-vertebrae, but does not show the repeating intervertebral disc staining characteristic of more proximal regions (Fig. 3B). These results suggest a deficit in chicken pygostyle sclerotome AP polarity.

To determine whether sclerotome AP polarity is aberrant in pygostyle development of other bird groups, PNA staining was conducted on rock dove (*Columba livia*, Fig. 3C) and emu (*Dromaius novaehollandiae*, Fig. 3D) embryos at HH36 equivalent stages. While the dove, a neoave (and neognath like the chicken), showed reduced and atypical distal PNA staining in the pygostyle region, the emu, a paleognath, showed a different pattern. Instead of preferentially staining intervertebral discs, PNA in the emu strongly stained the centra and neural arches of the cartilaginous prevertebrae, and no differences were observed between the pygostyle and free vertebrae regions. The distal tail distribution of the PNA lectin target is therefore disparate in different birds, and may indicate variation in sclerotome AP polarity in their respective groups. However, despite the variable patterns of PNA labeling, birds resegment somites correctly into sclerotomes and form separate vertebrae with intervertebral discs in the pygostyle region (Fig. 3E), later fusing their distal vertebrae into a pygostyle. These collective observations, in combination with the PNA link to the nervous system, suggest that while distal vertebrae fusion is not influenced by sclerotome polarity, neural crest-specific development within the pygostyle in different bird groups was worthy of further investigation. Neural crest, in conjunction with proper sclerotome AP polarity, is necessary for the segmented patterning of the nervous system along the axial column^40^.

### Peripheral nervous system and spinal nerve development

**Figure 4:**
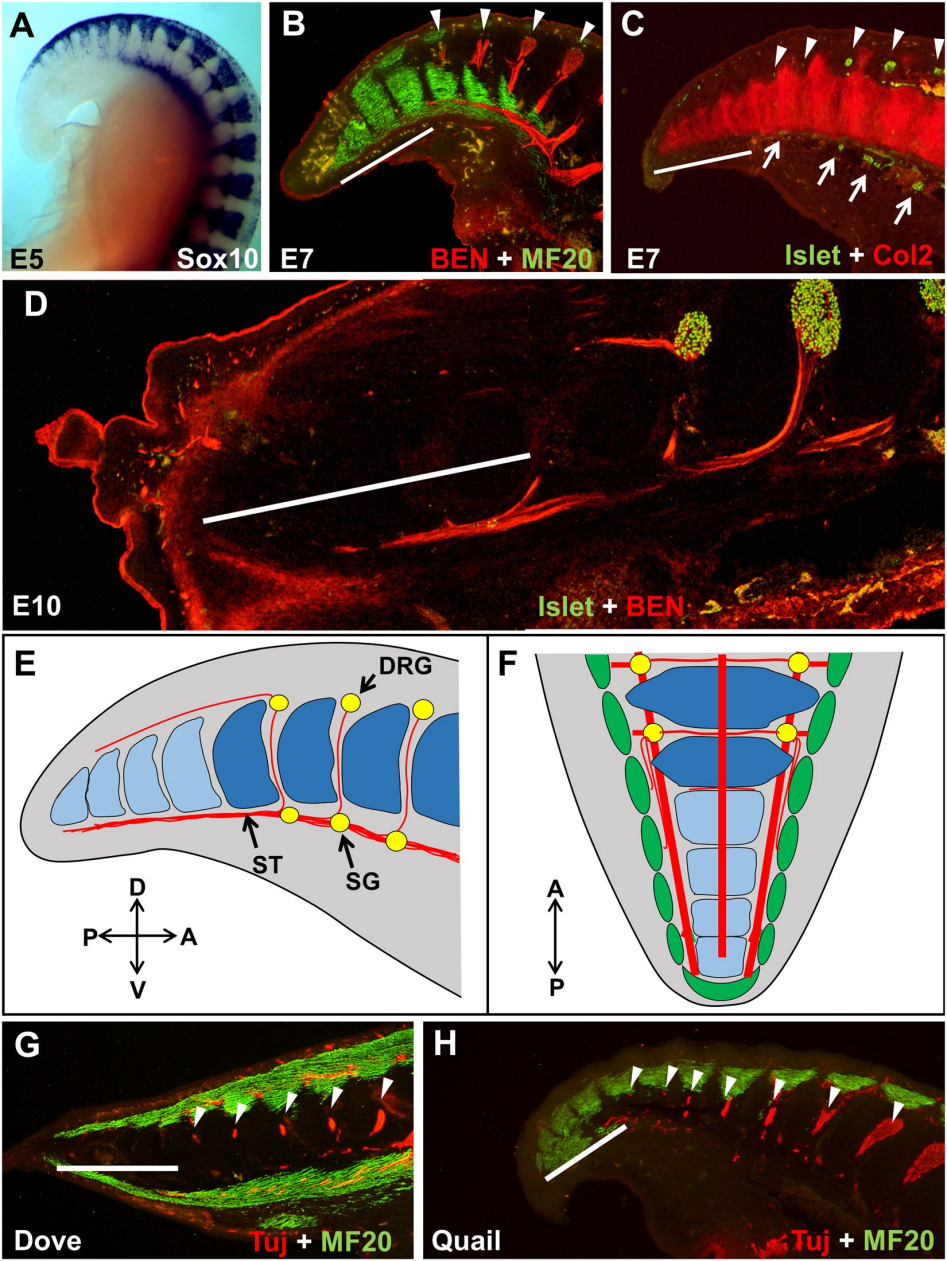
Tail peripheral nervous system development in chicken, dove, and quail. (**A-F**), chicken. (**A**) Sox10 ISH, stage E5, showing tapering neural crest development that terminates prior to the end of the tail. (**B**) E7 sagittal cryosection co-stained for neural tissue (BEN antibody, red) and myotome (MF20, green). DRG development (arrowheads) terminates one sclerotome level proximal to the pygostyle domain (white bar). (**C**) E7 sagittal cryosection co-stained for prevertebrae (Col2, red) and sensory neuron cell bodies (Islet 1/2, green). DRGs (arrowheads) and sympathetic ganglia (green) develop in register outside the pygostyle region, but not within. (**D**) E10 cryosection co-stained for DRGs (Islet1/2, green) and motor neurons (BEN, red). Motor neuron axons, which join the sympathetic tract, extend into the pygostyle region, to the distal end of the tail, beyond the distalmost development of DRGs. Dorsal nerve tracks emanating posteriorly from the distal DRGs also innervate the pygostyle region. (**E,F**) Schematics of chicken tail peripheral nervous system; (**E**) sagittal view; (**F**) dorsal view. Nervous system in red, pygostyle prevertebrae in light blue, free prevertebrae in dark blue, myotome in green, and ganglia in yellow. (**G**) Rock dove HH31 (equivalent to E7 in chicken), sagittal cryosection co-stained for nervous tissue (Tuj1, red) and myotome (MF20, green). Like chicken, the distalmost 4 pre-vertebrae lack DRGs (these are likely the pygostyle-specific prevertebrae, noted by white bar), and neural development is evident outside this domain. (**H**) Quail HH31 sagittal cryosection co-stained with Tuj1 (red) and MF20 (green). Quail distal neural development is consistent with chicken and rock dove, with a lack of DRG development in the pygostyle region (white bar). The diagrams in E and F are therefore representative of all three birds. Abbreviations: A: anterior; D: dorsal; DRG: dorsal root ganglia; P: posterior; SG: sympathetic ganglia; ST sympathetic tract; V: ventral. With the exception of F, all tails sagittal, with distal to the left.

The connection between somite/sclerotome polarity and spinal nerve development led to more thorough analyses of the neural system in the avian tail (we use the term spinal nerves to include the classic definition, just distal to the point where the dorsal and motor roots join, as well as the splitting to the dorsal and ventral rami). We questioned whether pygostyle-specific differences in sclerotome polarity in neognaths are reflected in nervous system development, specifically the formation of dorsal root ganglia (DRGs) and spinal nerves. We first analyzed early events in spinal nerve development relative to the pygostyle and free vertebrae domains in the chicken from E5 to E10. We stained chick embryo tails for neural crest (ISH, Sox10 probe), neural crest-derived neural cells (Islet 1/2 antibody), and motor neurons (BEN antibody), as well as for general neural tissue (Tuj 1 and anti-neurofilament antibodies). Collagen 2 and myosin heavy chain MF20 antibodies were employed to stain prevertebrae and dermomyotome/myotome, respectively. These results show that neural crest and its derivatives terminate one somite level anterior to the boundary between the pygostyle and free vertebrae regions (Fig. 4A-F). In the chicken embryo, DRGs and sympathetic ganglia were observed to terminate at the same sclerotome level (Fig. 4C). The ventral sympathetic tract, however, extends to the end of the tail, and its nerve fibers, which join the ventral roots, are seen to branch one sclerotome level posterior to the DRGs and sympathetic ganglia, still outside the pygostyle region (Fig. 4D).

The neural crest plays a pivotal role in spinal nerve development. Neural crest cells migrate to form the dorsal root ganglia that connect to the spinal nerves^41^, as well as the sympathetic tract and associated ganglia down the ventral axial column in vertebrate embryos^42^. Apart from contributing directly to peripheral nervous structures, the neural crest, in combination with somite polarity, is primarily responsible for establishing the segmented pattern of the axial peripheral nervous system. While the ventral roots of the spinal nerves originate in the neural tube^43^, the position of cell bodies of the roots is governed by boundary cap cells, which are another neural crest derivative^44,45^. Neural crest-derived boundary cap cells are also responsible for localizing the entrance points of sensory DRG neurons into the neural tube^46,47^. The alignment of sensory ganglia in the same dorsoventral plane as dorsal root ganglia is mediated by interactions between migrating neural crest, sclerotome, and neural tube^48,49^. Determining the posterior extent of neural crest, therefore, was a crucial consideration in correlating spinal nerve development with the pygostyle/free vertebrae boundary.

Previous studies did not address chicken neural crest development with respect to the pygostyle, but a lack of DRG formation in the distal tail has been observed and was found to be at least partly attributed to maintained expression of Noggin and concomitant loss of Bmp4 and Wnt1^50^. Neural crest cells form in reduced numbers the more posterior (as seen in Fig. 4A), but are also unable to delaminate, possibly due to the lack of the PNA target, and are depleted by apoptosis. Intrinsic to the neural crest cells from this axial level, the cells are only capable of differentiation to Schwann cells and glia, not to neurons^51^. These results, in combination with compromised sclerotome polarity, show that multiple processes are involved that prevent distal spinal nerve formation in the chicken.

Notably, we find that other neognath embryos, including rock dove and quail, also show a lack of spinal nerve development in the pygostyle region (Fig. 4G,H). Like the chicken embryo, the adult rock dove spinal nerves terminate one vertebra level anterior to the pygostyle^10^. These data show that spinal nerve development never initiates in the pygostyle domain in chicken, rock dove, or quail. Considering that adult neognathous pygostyles in general do not have potential exit foramina for spinal nerves^1^, we hypothesize the absence of distal spinal nerve development is a likely scenario in neognathous birds as a group.

**Figure 5:**
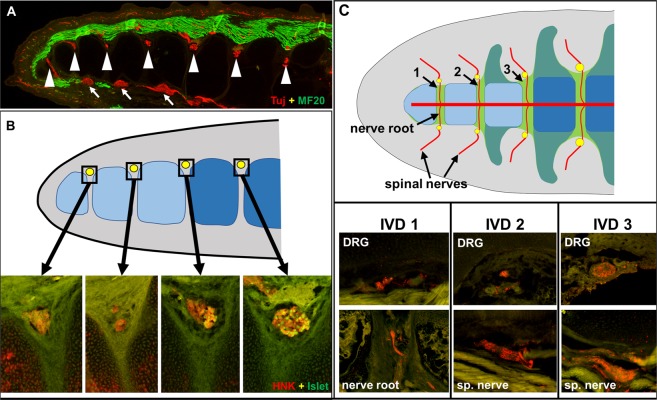
Tail peripheral nervous system in the emu embryo and hatchling. (**A**) HH36 equivalent stage cryosection, sagittal view (distal to the left), co-stained for nervous tissue (Tuj1, red) and myotome/musculature (MF20, green). Unlike chicken, dove, and quail, DRGs (arrowheads) and sympathetic ganglia (arrows) form in the pygostyle, to the very distal end of the tail. (**B**) Neural crest derivatives in the emu tail at HH36. Top, diagram of sagittal view indicating the axial level of DRGs, shown below. DRGs shown in yellow, pygostyle-specific prevertebrae in light blue, and free prevertebrae in dark blue. Below, DRGs (sagittal views) stained with HNK (red), indicating neural crest derivative, and Islet 1/2 (green), indicating neural crest-derived sensory neuron differentiation. Islet-positive cells are only observed outside the pygostyle (light blue in diagram), suggesting that despite development of neural-crest-derived DRGs, there is a lack of sensory neuron differentiation within the pygostyle. (**C**) Emu hatchling. Top panel, diagram, coronal/dorsal view; the distalmost intervertebral discs are labeled 1–3, posterior to anterior; IVDs in green, DRGs in yellow, nerve roots and spinal nerves as indicated; the central red bar denotes the spina cord. At this stage, the transverse processes of the vertebrae have not yet mineralized, and their cartilages are contiguous with the IVDs. Bottom panel, nervous system structures for each IVD as noted (IVD 1 and 2 are within the pygostyle; IVD 3 is outside the pygostyle); coronal cryosections stained with Tuj + RT97 (red) and overlayed on 488 nm background. The emu pygostyle domain is estimated from^1^ and from the MOR 186 specimen. Abbreviations: DRG: dorsal root ganglia, IVD: intervertebral disc, sp: spinal.

In the emu, foramina aligned with the spinal cord and intervertebral discs are observed in the bony pygostyle. This anatomical feature, coupled with the differences in PNA labeling, led us to investigate whether spinal nerve development follows a different pattern than in chicken, quail, and rock dove. At an equivalent HH36 stage (when terminal sensory neuron differentiation is well underway in other birds), DRGs, sympathetic ganglia, and spinal nerve roots were observed at the very end of the emu tail, to the level of the most distal sclerotome (Fig. 5A). In agreement with adult pygostyle morphology, therefore, emu spinal nerve development deviates from the other birds examined and instead continues into the pygostyle region. The disparate emu distal spinal nerve development indicates that the process of axial peripheral nervous system termination is not universal in avians.

While spinal nerve development was observed in the pygostyle, however, the emu pygostyle-specific DRGs do not appear to develop analogously to those in the free vertebrae region of the tail. HNK positive cells were detected in DRGs throughout the pygostyle domain, but Islet 1/2 positive cells were only detected in DRGs proximal to the pygostyle region (Fig. 5B). This suggests that while neural crest cells delaminate, migrate, and form DRGs in the emu pygostyle region, there is a pygostyle-specific loss or substantial delay of sensory neuron differentiation.

The observed lack of sensory neuron differentiation led us to question whether spinal nerves eventually form in the emu distal tail. To test for spinal nerves post-hatching, a hatchling emu tail was stained for neurofilament H (RT97) and neural β-III tubulin (Tuj 1). Nerve roots, DRGs, and spinal nerves were detected within the pygostyle (Fig. 5C). Despite an early lack of sensory neuron differentiation in the emu pygostyle DRGs, therefore, components of spinal nerves form, though further studies are required to determine if sensory inputs are generated and maintained. These nerves likely pass through the pygostyle foramina with blood vessels, observed by dissection to exit from the spinal channel (Supplementary Fig. 1).

**Figure 6:**
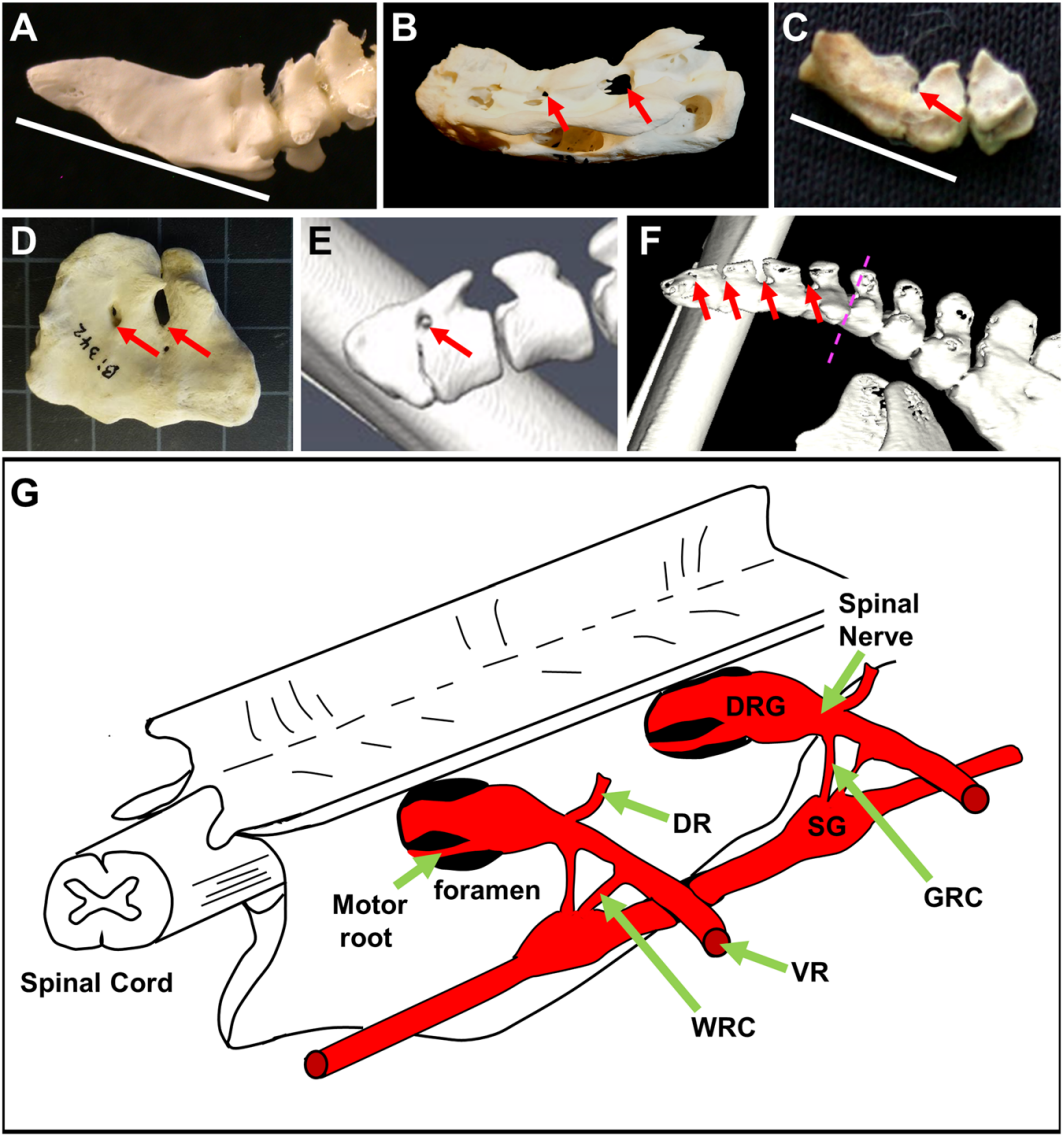
Potential exit points for spinal nerves and/or vasculature in paleognath pygostyles. (**A**) Juvenile chicken pygostyle (most proximal pygostyle vertebra in the process of fusing). Like other neognathous birds, the chicken pygostyle is solidly fused and smooth, with no foramina aligned with the spinal cord channel. White bar indicates pygostyle. (**B**) Adult emu (*Dromaius novaehollandiae*) pygostyle (MOR 186) (image also published in^1^). Foramina aligned with spinal cord channel noted by red arrows. (**C**) Adult elegant crested tinamou (*Eudromia elegans*) pygostyle (LACM 116227) (image also published in^1^). Incomplete zygapophyses fusion results in a crevice aligned with the spinal cord in the pygostyle, red arrow. (**D**) Adult ostrich (*Struthio camelus*) pygostyle (LACM Bi342). Two foramina, aligned with the spinal cord channel and resulting from incomplete zygapophyses fusion noted by red arrows. (**E**) Adult kiwi (*Apteryx mantelli*), female, 5 years old, microCT image. A foramina is maintained between the two fusing vertebrae of the pygostyle. (**F**) Adult kiwi (*Apteryx mantelli*), male, 14 years old, microCT image. In this older kiwi, two proximal tail vertebrae have fused into the synsacrum, and three more vertebrae have fused into the pygostyle (boundary noted by purple hashed line). Despite additional vertebral fusions, foramina/crevices are maintained in the pygostyle. (**G**) Schematic of generic avian synsacral fused vertebrae, showing exit foramina for spinal nerves, and peripheral nervous system configuration (adapted from^68^, pg. 247). Just lateral to the point where motor roots join the DRGs is the formation of the spinal nerves. The spinal nerves split into the dorsal and ventral rami; the ventral ramus connects to the sympathetic ganglia via the white and gray rami communicans. Abbreviations: DR: Dorsal Ramus; DRG: Dorsal Root Ganglion; GRC: Gray Ramus Communicans; SG: sympathetic ganglion; VR: ventral ramus; and WRC: white ramus communicans. All tails in sagittal view with distal to the left.

The presence of spinal nerves and exit foramina in the emu pygostyle raises the issue of whether the emu is unique, or whether it is a faithful representative of paleognathous birds for these traits. Due to the difficulty of obtaining other paleognathous embryos, we instead examined a number of adult paleognathous pygostyles. Unlike neognathous birds (Fig. 6A^1^;), the pygostyles in several paleognathous birds (emu, elegant crested tinamou, ostrich, and kiwi) demonstrate incomplete fusion of zygapophyses, resulting in either foramina or open crevices (Fig. 6B-F). These open spaces align with the spinal cord channel and intervertebral discs (before fusion), and are analogous to the exit points for spinal nerves and vasculature in more proximal regions (Fig. 6G). Spinal nerve development in other paleognaths, therefore, likely follows the emu pattern.

To address whether the open spaces/foramina are maintained during paleognath ontogeny, microCT analysis of 5- and 14-year old kiwi specimens was performed (Fig. 6E,F) (emus of multiple adult stages were not available for this study). Kiwis reach skeletal maturity at 5 to 6 years of age^52^, and these two specimens allowed us to sample early and later adult stages. In the younger specimen, the pygostyle consists of two distally fused caudal vertebrae, with seven proximal free vertebrae. In the older kiwi, two proximal caudal vertebrae have fused into the synsacrum, and the pygostyle incorporated four vertebrae. The total number of caudal vertebrae in both specimens is likely the same as chicken, amounting to nine. The greater number of incorporated pygostyle and synsacrum vertebrae in the older specimen is likely due to progressive vertebral fusion (though individual variation cannot be discounted). Despite additional vertebral fusion, potential exit foramina for spinal nerves and/or vasculature are maintained, similar to what we have observed in the emu, in which foramina are present throughout the adult pygostyle. In a 1.5 year old chicken^1^, its pygostyle was equivalent in both size and dimension to pygostyles of 6 month specimens, suggesting that progressive pygostyle fusion beyond initial pygostyle formation is not universal in avians. Different strategies may exist, therefore, to preserve distalmost spinal nerves and/or vasculature.

**Figure 7:**
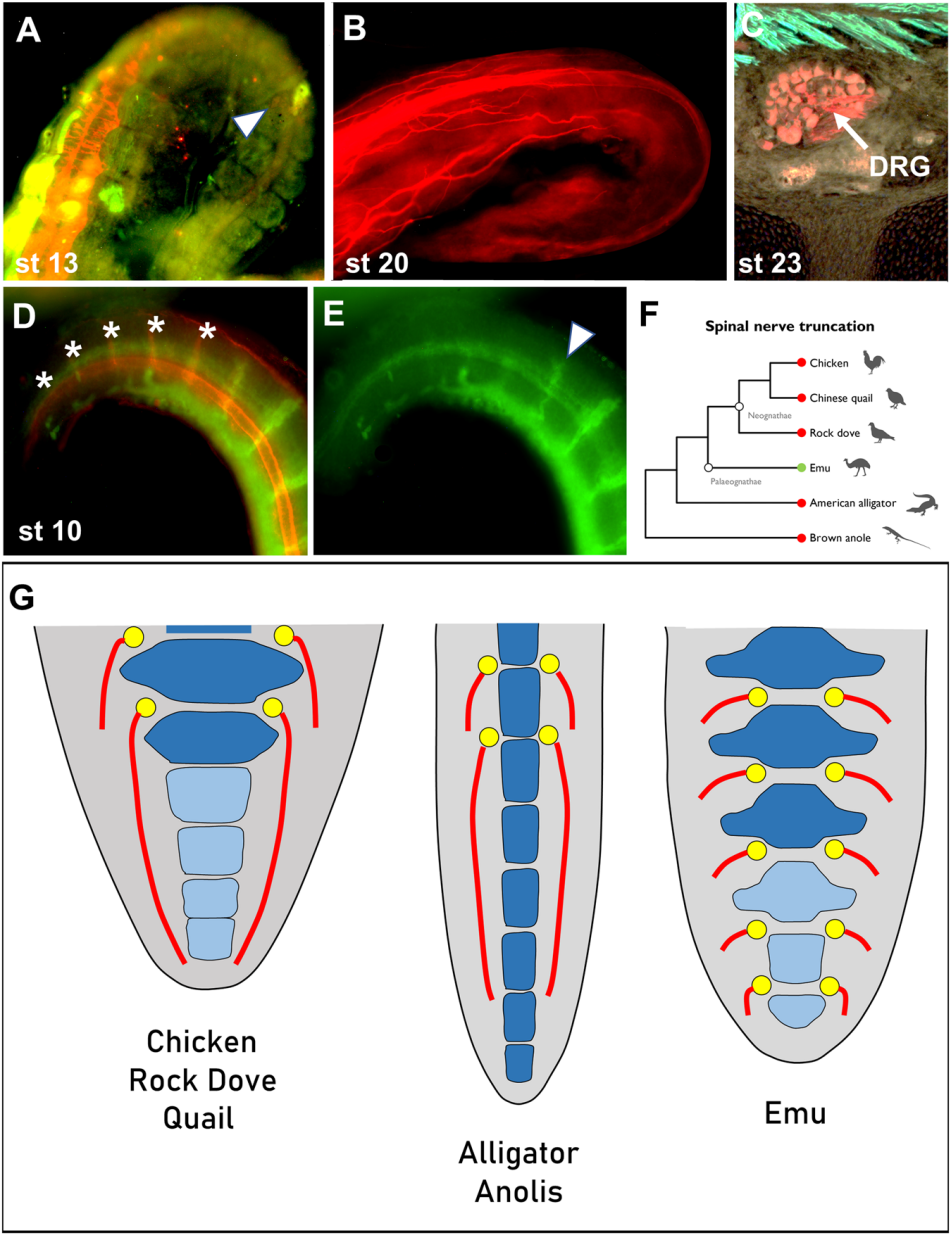
Diapsid spinal nerve development. (**A-C**) Alligator (*Alligator mississippiensis*) embryo spinal nerve development. (**A**) Stage 13 alligator embryo, wholemount, co-stained for neural tissue (Tuj 1, red) and myotome (MF20, green) (tail curved to the right; tail tip at the bottom right). Neural development is observed at the left, proximal side of the curve, but lacking at the right, distal end of the tail; white arrowhead denotes posteriormost level of neural crest, out of view). (**B**) Stage 20 alligator embryo, wholemount, stained for neural tissue with Tuj 1 antibody. The spinal cord extends to the end of the tail, but spinal nerve branches terminate significantly before the tail terminus. (**C**) Stage 23 alligator embryo cryosection, co-stained for neural tissue (Tuj 1) and muscle tissue (MF20). The most distal DRG was observed between the 6th and 7th vertebrae from the end of the tail, consistent with earlier stages. (**D,E**) Stage 10 anole (*Anolis sagrei*) wholemount embryo. (**D**) Embryo tail co-stained for intervertebral discs (Col2, red; noted by asterisks) and neural tissue (Tuj 1, green). (**E**) Same embryo tail as in D, showing only Tuj 1-specific 488 nm fluorescence. The most distal DRG is observed between the 6th and 7th most distal prevertebrae, similar to alligator. (**F**) Cladogram of the diapsids examined in this study, showing emu as the only tested diapsid with DRG formation extending to the end of the tail. (**G**) Diagram comparing dorsal views of the diapsids examined in this study. Chicken, quail, and rock dove lack spinal nerve development in their distal tails, as do alligator and Anolis. Emu is unique, with DRGs and eventual spinal nerves forming to the tail terminus. Light blue elements: pygostyle-specific; dark blue: free vertebrae; yellow: DRGs; red: spinal nerves.

Because emu distal nerve development differs substantially from quail, rock dove, and chicken, and as a paleognath the emu represents a more basal avian group, we sought to determine whether the emu follows a more ancestral distal spinal nerve configuration. The configuration of Mesozoic dinosaur distal spinal nerves is unknown, but the ancestral state can be hypothesized in the context of the fossil record and living archosaurs. The most conservative scenario is that all modern birds descended from a single long-tailed ancestor. That ancestor incurred mutations that shortened and fused the tail, which led to a radiation of birds with short, fused tails in the Early to Mid- Cretaceous, giving rise to the Pygostylia group^2^. Since emu distal spinal nerve development differs from neognaths, its anatomy may reflect a more basal condition, and would instead mirror that of long-tailed Crocodilia, the closest archosaurs to avians. To test this hypothesis, stage 13^53^ alligator embryo tails were immunostained in wholemount for neural tissue and myotome/muscle. Surprisingly, like chicken, rock dove and quail (but unlike emu), alligator embryos lack distal spinal nerve development (Fig. 7A-C). Spinal root development was absent in the six distal sclerotomes at the tail tip. Older alligator embryos at stage 20 (Fig. 7B) and stage 26 (Fig. 7C and Supplementary Fig. 2) were also analyzed by immunostaining, and the distal spinal nerve anatomy was consistent with the earlier stage. To determine whether other long-tailed reptiles share this morphology, brown anole embryos at stage 10^54^ were similarly immunostained (Fig. 7D,E). Like alligator, chicken, rock dove and quail, brown anole embryos lack spinal nerve formation at the tail tip; no spinal nerve development was observed alongside their distalmost six sclerotomes. From these data, we hypothesize that the ancestral configuration is an absence of spinal nerve development at the distalmost end of the tail. If our hypothesis is correct, the emu does not follow this proposed ancestral pattern (Fig. 7F). The overall process at issue here is axial termination, and disparities at the tail tip across vetebrates indicate variations in how the different species terminate their spinal columns. The extension of spinal nerves to the distal end of the tail into the pytostyle in the emu differs from the other species tested, suggesting several possible evolutionary scenarios that could account for this divergence.

### Evolutionary considerations

Our data points to several possible scenarios during bird evolution that could have given rise to the alternative distal spinal configuration in emus compared to other birds and reptiles (Fig. 7G and Supplementary Fig. 3). One possibility is that the anatomy of distal spinal nerves is highly variable, with no discernible pattern, and our sample size is too small to reveal this situation. Another possibility is that emus, or paleognaths as a group, sustained mutational change(s) that caused the extended distal boundary of neural crest, and subsequent spinal nerve formation to the very end of the tail. A third scenario is that evolutionary events in early birds resulted in an emu-like morphology, with caudally extended neural crest and spinal nerves extending into the pygostyle. This scenario implies that emus reflect the more basal state, and neognathous birds incurred additional mutations that led to the distalmost absence of spinal nerves. In this case, the loss of distal spinal nerves in neognathous birds arose from a multi-step set of events. Our finding that emus form two more caudal spinal nerves than chicken, quail, and rock dove is compatible with this hypothesis, though additional vertebra loss would have occurred in emus. These first three scenarios are consistent with a single long-tailed avian ancestor. The fourth possibility is that emus, and perhaps the Paleognathae group as a whole, evolved from a different Mesozoic long-tailed ancestor than neognathous birds. In this case, the tails of emus and/or other paleognaths shortened and distally fused in separate, convergent events from other birds. Evidence promoting any one of these scenarios awaits further fossil discoveries; evo-devo approaches will also likely help to determine whether individual traits are synaptomorphic or convergent (please see Supplementary Fig. 3 for more detailed discussion of these hypotheses).

The phylogenetic split between paleognaths and neognaths^55,56^ is predicted to have occurred in the Cretaceous period, with estimates ranging from approximately 73 million^56^ to 110 million years ago^57^. In that timeframe, feathered theropods existed worldwide, and avians had long since branched from non-avian theropods. The approximately 120 million year old fossil specimens from the Jehol biota reveal that long- and short-tailed avialans lived simultaneously^5^. At that time and place, specific groups of short-tailed avialans have been identified, within the Pygostylia group, and a recent evaluation of their pygostyles shows a similar variation in morphologies as exists in modern birds^58^. Emu-like foramina have also been seen in confuciusornithid pygostyles, indicating that the emu distal spinal nerve configuration may be rooted in very early bird origins^1,58^.

Currently, it is unresolved whether the different Mesozoic Pygostylia subgroups all derived from a single long-tailed ancestor. In addition, a shortened tail with a distally fused pygostyle is known to have arisen independently in a non-avian dinosaur^59^. This finding, in combination with (1) our observation that one of the most common anomalies in otherwise viable avian embryos is tail defects, (2) several modern bird species have sustained additional tail truncating/distal fusion but otherwise viable mutations (i.e. Rhea and rumpless chicken breeds), (3) an early avialan has recently been described with a pygostyle but with features more basal than the long-tailed *Jeholornis*^60^ and (4) approximately one-third of known mouse mutations that cause caudal vertebral fusion also cause tail truncation^8^, suggests that independent tail truncation and pygostyle formation are not unprecedented, and could have occurred in more than one ancestral avian taxa. Also, it has been proposed that differences in pygostyle morphologies and phylogeny-specific evidence of rectricial bulbs in Cretaceous avians support convergent tail evolution among early Pygostylians^58^. Birds gained evolutionary advantages with short, distally fused tails, both for flight and for sexual selection^61,62^. Since more than one species of long-tailed, flighted maniraptorans have been identified, it is not unreasonable to hypothesize that the Pygostylia group arose from more than one long-tailed ancestor, and that descendants of different branches have survived into modern times.

In conclusion, emus, and likely paleognaths as a group, manifest unique spinal nerve configuration in their tails compared to representatives of neognaths. The disparate spinal nerve patterns are consistent with their respective embryonic development as well as with their adult anatomies. These results, in combination with spinal nerve formation in alligator and brown anole that mirrors the neognath pattern, suggest several possible evolutionary scenarios, including the possibility of an independent paleognath long-tailed ancestor. While interesting patterns are emerging that suggest divergent evolutionary paths, future studies involving more representatives of the three modern avian groups are needed to substantiate the sclerotome polarity and spinal nerve development variation between bird groups. Overall, the differences between paleognaths and neognaths, as well as their similarities, are building a more comprehensive picture of avian evolution.

## Methods

### Animals

#### Chicken

Staging was according HH stages^63^, or Embryonic (E) days. Except for peanut agglutinin staining, fertilized white leghorn chicken eggs were obtained from Charles River Laboratories (Illinois), and used for studies up to E15. Fertilized eggs for peanut agglutinin-staining were obtained from a local farm, and were of mixed *Gallus domesticus* breeds.

For E17 embryos, and D8 hatchings, fertilized bovan brown chicken eggs were sourced from Clemson University, and incubated and harvested as detailed below.

#### Quail

Fertilized Chinese painted quail (*Coturnix chinesis*) eggs were obtained from an online breeder, and incubated until the desired level of development. A development chart is not available for this species, but they appear to develop in the same timeframe as Japanese quail^64^. Embryos were harvested at HH31 (E6.5) and HH36 (E8.5), and processed as described below for chicken eggs.

#### Rock dove

Fertilized homing rock dove (*Columba livia*) eggs were obtained from a local Montana breeder, and incubated as above. Developmental stage for harvesting was assessed according to^65^.

#### Emu

Fertile emu (*Dromaius novaehollandiae*) eggs, incubated to HH36 (E21^66^;) were obtained from the Montana Emu Ranch (Kalispell, MT), as well as frozen carcasses of post-hatch birds.

#### Kiwi

Two north island kiwi (*Apteryx australis*) specimens (expired from natural causes), a 5.33 year old female and a 12.3 year old male, were obtained by Holly Woodward through arrangements with Kathleen Brader and Zoo Zlin-Lesna (U.S. Fish and Wildlife Service import permit number 130066 and Czech Republic, respectively). The specimens were frozen after necropsy, which included thoracoabdominal organ removal. Upon thawing both specimens were immediately fixed in 10% neutral buffered formalin for 28 days.

#### Alligator

Fertilized American alligator (*Alligator mississippiensis*) eggs were obtained by the Parrott and Chuong laboratories, from the Yawkey Wildlife Center in Georgetown, SC (permit #SC-06-2018) and the Rockefeller Wildlife Refuge in Louisiana, respectively. Eggs were incubated at 30 °C and the embryos harvested at the appropriate level of development according to^53^. Embryos were fixed in either 4% PFA or Dent’s fixative (80% MeOH, 20% DMSO).

#### Brown anole

*Anolis sagrei* embryos were obtained by Douglas Menke from his University of Georgia Athens breeding colony. Developmental stage was gauged according to^54^. Embryos were fixed in Dent’s fixative.

#### Museum specimens

Ostrich (*Struthio camelus*, LACM Bi342) and elegant crested tinamou (*Eudromia elegans*, LACM 116227) pygostyles were photographed from the LA County Natural History Museum (Los Angeles, CA) ornithology collection. The adult emu pygostyle (MOR 186) was photographed from the collections of the Museum of the Rockies (Bozeman, MT).

Except for emu eggs (which were incubated at the Montana Emu Ranch), all other bird eggs were incubated at 38 °C with automatic egg turning until the desired level of development. All embryos (bird and reptile) were harvested into PBS and fixed with either 4% PFA (4 °C) for two to three days, or Dent’s fixative (−20 °C) overnight.

All embryo manipulations adhered to institutional guidelines and were approved by the Institutional Animal Care and Use Committees at Montana State University (IACUC, protocol #2018-82), Clemson University (AUP #2019-047), University of Southern California, and University of Georgia (AUP #A2017 12-004). Appropriate approvals were also obtained from the South Carolina Department of Natural Resources and the Louisiana Department of Wildlife and Fisheries.

### Fluorescent labeling

For immunofluorescence performed on cryosections, embryo tails were cryoprotected overnight in 30% sucrose in 0.1 M Sodium Phosphate buffer pH 6.3, then infiltrated and cryoembedded in OCT medium (TissueTek). Fourteen to 20 µm sections were collected and applied to either Superfrost Plus (Fisherbrand) or agar-coated glass slides. All sections were blocked for 1 hour in NGS blocking buffer (0.03 M Tris pH 7.5, 0.15 M NaCl, 1% glycine, 0.4% Triton X 100, 10% goat serum) prior to antibody incubation. Presomitic mesoderm, undifferentiated somites, dermomyotome and dermatome were imaged with Pax7 antibody (Developmental Studies Hybridoma Bank (DSHB) and R&D Systems). Myotome was detected using the MF20 mAB (DSHB), specific for sarcomeric myosin. Neural crest derivatives were detected using the HNK1 antibody (DSHB), as well as the DRG/sensory neuron-specific antibody, islet 1/2 (DHSB). BEN mAb (DSHB) was used to image motor neurons and axial-specific nerve tracts in chicken embryos. Tuj1 mAB (anti-β-III tubulin, R&D Systems) and RT97 mAB (anti-neurofilament H, DHSB) were employed for pan-neural staining. Collagen II mAb (DSHB) was used as a marker for early intervertebral discs (E5-E6), notochord (E5-E7), and cartilaginous prevertebrae (E7-E11). All concentrated DSHB antibodies were used at 1:100, and all cell supernatants were used at 1:20 dilution. Pax7 (R&D Systems) was used at 1:100 dilution. All antibodies were diluted in NGS and incubated overnight at 4 °C. Double staining was achieved using isotope-specific Alexa fluorophore labeled (488, 568 or 594 nm) secondary antibodies (Molecular Probes). High levels of autofluorescence in the emu hatchling required H_2_O_2_ treatment (6% diluted in PBS) for one min prior to NGS blocking. Later stage emu (hatchling) and alligator (stage 23) tails required decalcification by EDTA prior to embedding. For immunofluorescence performed on wholemount tissue, alligator and anole embryo tails fixed in Dent’s fixative were treated with hyaluronidase (Sigma; 2 mg/ml in PBS) for 3 hr at 37 °C), blocked in NGS overnight, treated with primary antibodies (Tuj1, MF20, Col2) for 7 days at 4 °C, and treated with secondary antibodies for 3 days at 4 °C subsequent to dehydration by an EtOH series followed by clearing by Benzyl Alcohol/Benzyl Benzoate.

Phalloidin visualization of somites was achieved with Actistain 488 phalloidin (Cytoskeleton) in wholemount tissue and on cryosections. E4-E6 embryos were fixed in 4% PFA. Wholemount E5 and E6 embryo tails were permeabilized with 0.5% Triton-X 100/PBS for 2 hr at room temperature and 2 mg/ml hyaluronidase (Sigma) in PBS for 1.5 hr at 37 °C. Cryosections were also permeabilized with 0.5% Triton-X 100 prior to phalloidin incubation, for 10 min at room temperature. Phalloidin staining on wholemount tissue and cryosections was performed at 0.25 μM in PBS, for 3 days at 4 °C, or 40 min at room temperature, respectively.

Peanut agglutinin staining was performed on cryosections. Sections on slides were treated with 0.5% cetylpyridinium chloride (in 0.1 M sodium phosphate butter pH 6.3) for 40 min at 37 °C. The sections were then blocked in HBS (10 mM HEPES, 0.15 M NaCl, 0.1 mM CaCl2, 1% BSA, 1% goat serum), followed by incubation in 10 µg/ml biotinylated peanut agglutinin (Vector Labs) in HBS for 40 min at room temperature. After washing, the slides were incubated in 5 µg/ml streptavidin Alexa 568 (Molecular Probes) for 1.5 hr at room temperature before washing and mounting.

DiI labeling of embryo vasculature was performed as described^67^. Briefly, DiI (I,I′-dioctadecyl-3,3,3′,3′-tetramethylinocarbocyonine perchlorate, Sigma) was reconstituted in DMSO, then diluted to 4 mg/mL in 0.3 M sucrose, and microinjected into vitelline arteries of E5-E7 embryos in ovo. The injected embryos were returned to the egg incubator for 10 min, and subsequently harvested into PBS and fixed in 4% PFA. Injected embryos were then processed for cryoembedding and sectioning, and subsequent immunofluorescence staining.

Imaging of cryosections and wholemount tissue was performed on a Zeiss Axioscope A.1 microscope in conjunction with a Jenoptik ProgRes C14 Plus digital camera and accompanying software.

### TUNEL labeling and *in situ* hybridization (ISH)

TUNEL labeling for cell death was carried out according to standard protocols. Briefly, E5 embryos were fixed in Dent’s fixative, and bleached with 6% H2O2 in MeOH. Embryos were subsequently rehydrated through a series of MeOH to PBS, before proteolysis in 10 µg/mL proteinase K for 10 min at room temperature. The DIG-dUTP end-labeling reaction (0.5 µM DIG-dUTP (Roche/Sigma), 250 U/mL TdT enzyme (Roche/Sigma)) proceeded overnight at room temperature. Following wash steps, labeled embryos were incubated overnight in anti-DIG-AP antibody (1:1000, Roche/Sigma), and developed in BCIP/NBT solution (Millipore). *In situ* hybridization (ISH) was performed according to the Geisha website protocols (www.geisha.arizona.edu). The Sox10 probe construct, Sox10 pGEM-T, was generated by PCR amplification of the Sox10 gene from HH20 random-primed chick embryo cDNA. Sox10 PCR primers were GGGGATCCCTCATTTCATAGCCCGTATGTGTC (forward) and GAGGACAGGGCTCAAATAGGTTAC (reverse), which amplifies a 905 nucleotide fragment (sequence ID NM_204792.1). The amplified product was verified by sequencing (University of Montana Murdock DNA Sequencing Facility) and subcloned into the pGEM-T Easy vector (Promega). Pax1 and Pax9 ISH probe constructs were generated by DNA synthesis and cloning into pUC19 (Genscript). The Pax1 pUC19 construct contains the Gallus gallus Pax1 sequence corresponding to nucleotides 476–948 of Genbank Seq. ID XM_015283428, subcloned into 5′ EcoR1 and 3′ Pst1 restriction sites. The Pax9 pUC19 construct contains the *Gallus gallus* Pax9 sequence corresponding to nucleotides 247 to 524 of Genbank Seq. ID NM_204912.2, subcloned into 5′ EcoR1 and 3′ BamH1 sites.

### Histology staining

Alcian blue and alizarin red staining on E17 wholemount embryos was performed as previously described^1^. Alcian blue and picrosirius red histology staining for chicken E11 and D8 stages was performed on paraffin embedded sections (5 µm); the chicken D8 tails were first decalcified by EDTA prior to embedding. Briefly, sections were rehydrated through a xylenes to ethanol series, followed by 15 minutes incubation in Alcian blue (0.048% Alcian Blue 8GX (Alfa Aesar), 70% EtOH, 20% glacial acetic acid), running water wash, and then 1 hr incubation in picrosirius red (0.1% Direct Red 80 (Sigma) in saturated aqueous picric acid). After a brief wash in acidified water, the slides were dehydrated and mounted in DPX medium (Sigma). Hematoxylin staining on E6 chicken embryo cryosections was performed by a 1 min incubation in Gill’s Hematoxylin #2 (Polysciences). After washing, the slides were dehydrated through an ethanol and xylenes series, and mounted in DPX medium.

### MicroCT scanning and imaging

The kiwi specimens were microCT scanned to image skeletal anatomy on a 2013 Nikon 225 XT H microcomputed tomography system (Nikon Corp., Shinagawa, Tokyo, JPN). For all scans the samples were double-wrapped in heat-sealed polyethylene bags to prevent dehydration during scanning and physically stabilized using polyethylene foam within a plastic mounting unit. Skeletal data for the male was collected at 107.9 microns resolution (isometric voxels), using 150 kilovolts (kV), 61 micro-amperages (μA), 708 millisecond (ms) exposure timing, no multi-frame averaging, without a filter, and on a tungsten reflection target in two sections for 74 min. Skeletal data for the female was collected at 104.5 microns resolution, using 150 kilovolts (kV), 61 micro-amperages (μA), 708 millisecond (ms) exposure timing, no multi-frame averaging, without a filter, and on a tungsten reflection target in two sections for 74 min. The two resulting TIFF image stacks for each individual were fused to produce a complete male and complete female sample, using the “3D Stitching” feature of ImageJ (National Institutes of Health, Bethesda, Maryland, USA).

## Supplementary information

Supplementary information.
