# Supplementary material for: Distal spinal nerve development and divergence of avian groups
Source: Sci Rep. 2020 Apr 14;10:6303. doi: 10.1038/s41598-020-63264-5 (PMC7156524; doi:10.1038/s41598-020-63264-5)
Supplement: Supplementary file 1 — Supplementary information. [file 41598_2020_63264_MOESM1_ESM.docx]

**Supplementary File**

**Distal spinal nerve development and divergence of avian groups**

**Authors/Affiliations:**

Dana J. Rashid^1,2^*, Roger Bradley^1^, Alida M. Bailleul^3^, Kevin Surya^4^, Holly N. Woodward^5^, Ping Wu^6^, Yun-Hsin (Becky) Wu^6^, Douglas B. Menke^7^, Sergio G. Minchey^7^, Ben Parrott^8^, Samantha L. Bock^8^, Christa Merzdorf^1^, Emma Narotzky^9^, Nathan Burke^10^, John R. Horner^11Ɨ^, and Susan C. Chapman^10Ɨ^

* Corresponding author

^Ɨ^These authors contributed equally

^1^ Department of Cell Biology and Neuroscience, Montana State University, Bozeman, MT 59717, USA; ^2^ Department of Microbiology and Immunology, Montana State University, Bozeman, MT 59717, USA; ^3^ Key Laboratory of Vertebrate Evolution and Human Origins, Institute of Vertebrate Paleontology and Paleoanthropology, Chinese Academy of Sciences, Beijing 100044, China and CAS Center for Excellence in Life and Paleoenvironment, 100044 Beijing, China; ^4^ Honors College, Montana State University, Bozeman, MT 59717, USA; ^5^ Department of Anatomy and Cell Biology, Oklahoma State University Center for Health Sciences, Tulsa, OK 74107, USA; ^6^ Keck School of Medicine, University of Southern California, Los Angeles, CA 90033, USA; ^7^ Department of Genetics, University of Georgia, Athens, GA 30602, USA; ^8^ Savanah River Ecology Laboratory and Odum School of Ecology, University of Georgia, Athens, GA 30602, USA; ^9^ American Studies Program, Montana State University, Bozeman, MT 59717, USA; ^10^ Department of Biological Sciences, Clemson University, Clemson, SC 29634, USA; ^11^ Honors Program, Chapman University, Orange, CA 92866, USA

**Glossary of Terms**

***Chondrogenesis*:** formation of cartilage.

***Convergent*:** from an evolutionary perspective, refers to similar traits that evolve independently

***Dermatome*:** somite compartment derived from the separation of dermomyotome (to dermatome and myotome); gives rise to skin.

***Dermomyotome*:** somite compartment that arises from the first compartmentalization (with sclerotome); gives rise to muscle and skin.

***Diapsid*:** an amniote tetrapod within the group that incudes birds/dinosaurs, crocodilians, lizards, and snakes.

***Intervertebral discs*:** cartilaginous tissue between vertebrae, derived from sclerotomes and notochord.

***Lectin*:** carbohydrate binding protein, with a high specificity for particular sugar groups. For this study, a commercial lectin isolated from peanuts was utilized to differentiate between the anterior and posterior haves of sclerotomes, which (usually) produce unique sugar moieties.

***Maniraptorans*:** (clade Maniraptora), theropod dinosaurs that include the subgroups Avialae (which includes modern birds)*,* Deinonychosauria, Oviraptorosauria, and Therizinosauria. All subgroups were evident in the Jurassic, but only Avialae survived the Kpg extinction event.

***Myotome*:** somite compartment derived from the separation of dermomyotome (to dermatome and myotome); gives rise to muscle.

***Neognathae*:** one of two groups of modern birds, and includes the **galloanseriforms** (e.g. chickens, ducks, quail), and **neoaves** (largest branch of modern birds, including hawks, owls, hummingbirds, penguins, etc.).

***Neural crest*:** embryonic migratory tissue derived from the dorsal neural tube that gives rise to the peripheral nervous system (among other structures).

***Neural tube*:** embryonic structure that is the precursor of the brain and spinal cord; gives rise to the central nervous system and the motor roots of the spinal nerves. The neural tube is formed by both primary neurulation, in which a neural plate forms a neural tube along the anterior-posterior axis of an embryo to the end of the trunk, and by secondary neurulation, where a solid core of cells cavitates to become a tube at the caudal end of the embryo. Secondary neurulation in mammals is negligible, such that the spinal cord and spinal nerves are all formed from only primary neurulation, and the spinal cord terminates in the lumbar region, coinciding with the point of primary neurulation termination. Secondary neurulation in birds and reptiles, however, is robust, and accounts for the continuation of the spinal cord and spinal nerves caudal to the trunk/hindlimb.

***Notochord*:** rod-like embryonic structure ventral to the neural tube that gives rise to the nucleus pulposus of the intervertebral discs and is an important signaling center in the embryo.

***Paleognathae*:** one of two groups of modern birds, and includes cassowaries, emus, kiwis, ostriches, rheas, and tinamous.

***Pleiotropic*:** a pleiotropic condition is one in which more than one phenotype arises from a single genetic mutation.

***Polarity*:** axis-specific asymmetric orientation. Polarity in a somite, for example is demonstrated in the anterior/posterior axis and dorsal/ventral axis, leading to 4 somitic regions.

***Presomitic mesoderm*:** mesoderm flanking the neural tube in an embryo that differentiates into somites.

***Pygostyle*:** compound bone at the end of an axial column formed by the fusion of the distal caudal vertebrae. This structure is found in all modern birds, in Cretaceous Pygostylia avialans, and at least one non-avian Mesozoic dinosaur.

***Resegmentation*:** in somite maturation, somites divide into rostral and caudal segments, and the caudal half of one sclerotome fuses with the rostral half of the next sclerotome; the boundary tissue in the center of the newly formed resegmented sclerotome gives rise to **von Ebner’s fissures** and then to intervertebral discs. Resegmentation aligns myotomes with intervertebral disc spaces for eventual axial muscle vascularization, neural innervation, and appropriate attachment to vertebrae.

***Sclerotome*:** ventralmost somite compartment after the first compartmentalization event (giving rise to sclerotome and dermomyotome); vertebrae and intervertebral discs are derivatives of this compartment.

***Somites:*** paired clusters of epithelialized cells, on either side of the neural tube, along the axial column of an embryo. They are the first developmental origins of vertebrae. **Somitogenesis** is the process of forming somites.

***Somite segregation*:** separation of individual somites from the presomitic mesoderm.

***Synaptomorphic*:** inherited from a common ancestor.

***Synsacrum*:** compound skeletal structure in the pelvic region of birds, some dinosaurs and pterosaurs resulting from the fusion of multiple thoracic, lumbar, sacral and proximal caudal vertebrae. The synsacrum fuses into the pelvic girdle in modern birds, resulting in a substantial supportive contribution to the skeleton.

***Tailbud mesenchyme*:** undifferentiated population of mesenchymal cells at the caudal end of the embryonic axial column following gastrulation that gives rise to the secondary neural tube, tail gut, somites and their derivatives in the tail.


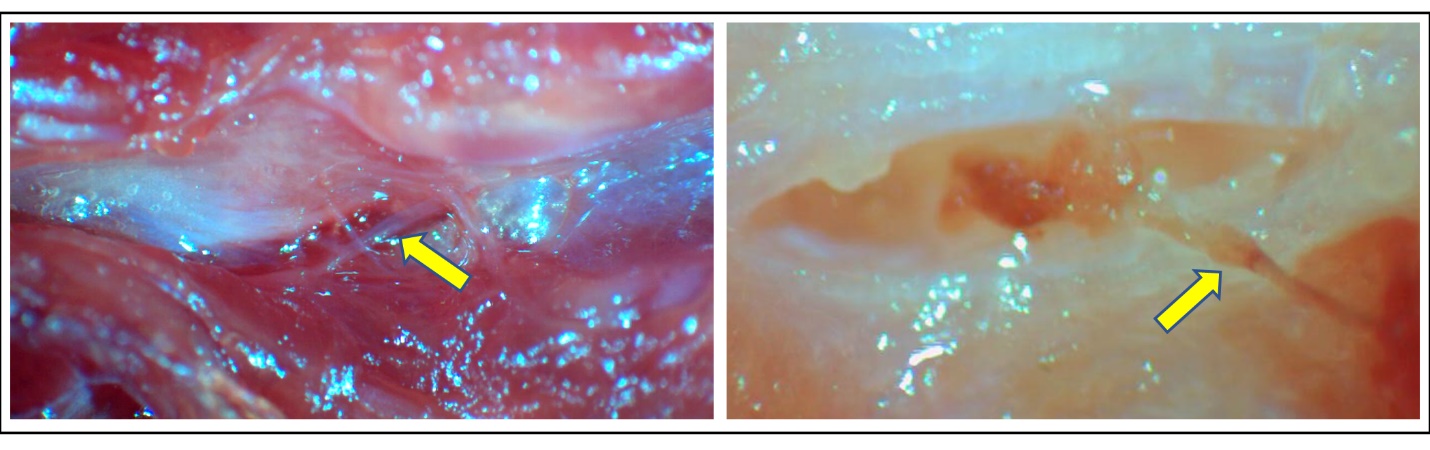


**Supplementary Figure 1. Dissection of a 5.5 month old emu, showing exit of blood vessels through pygostyle foramina.** Left panel, distal pygostyle foramen (partial dissection), sagittal view; yellow arrow indicates blood vessel. Right panel, proximal pygostyle foramen, sagittal view, with blood vessel pulled free of tissue.


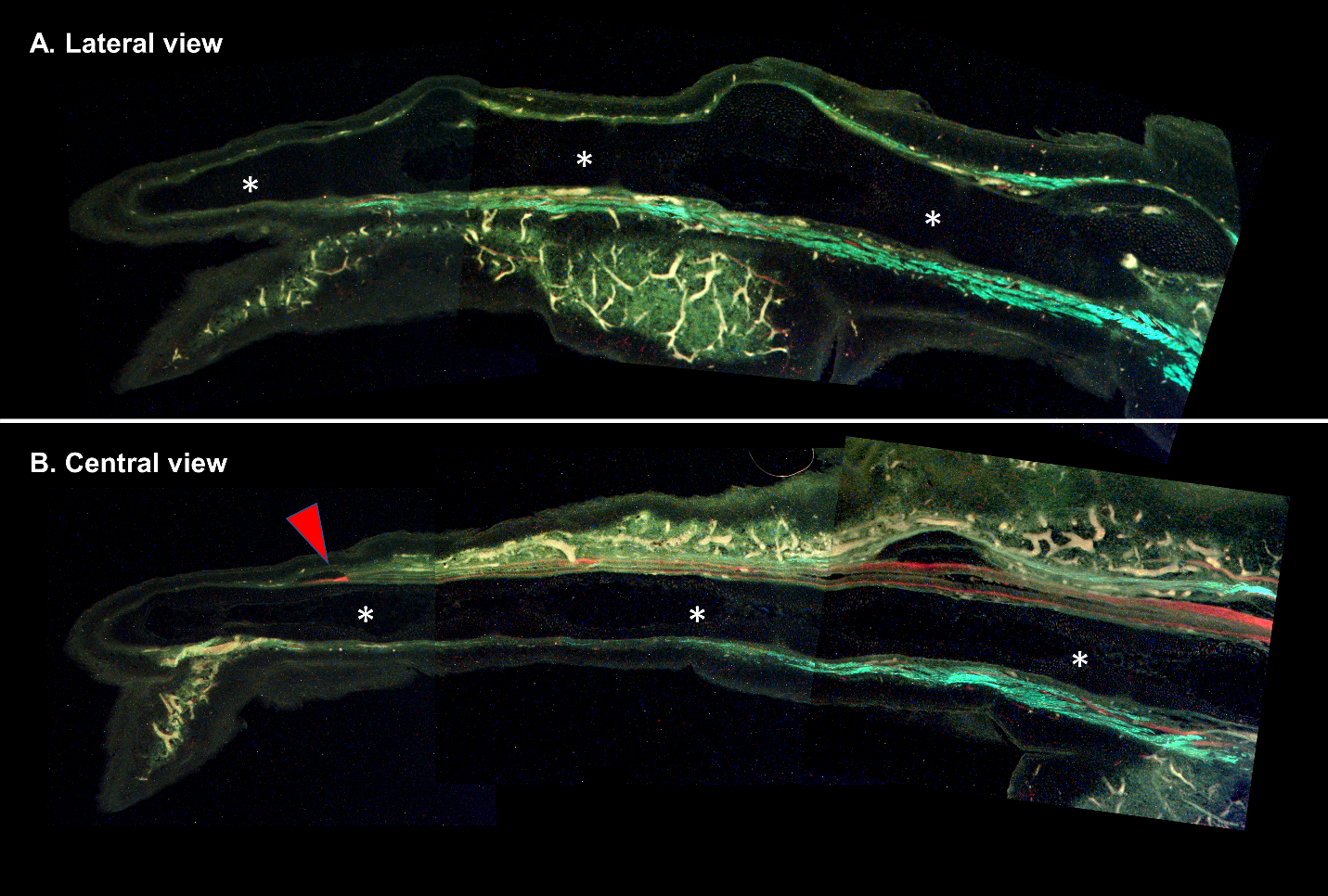


**Supplementary Figure 2. Lack of spinal nerves in the distal tail of a stage 26 (E46) alligator embryo.** Both images distal to the left; co-stained for neural tissue (Tuj1, red) and sarcomeric myosin (MF20, green). Asterisks indicate position of intervertebral discs. Individual images were taken at 5X, then stitched together in Photoshop for the final composite images. **A.** Sagittal view, just lateral to the spinal cord/midline. In more proximal regions, DRGs and spinal ganglia would be expected in this approximate plane in more proximal regions, but are not found in the distal tail. **B.** Sagittal view, through the spinal cord/midline. The spinal cord is found to persist posteriorly to the most distal pre-vertebra, but spinal nerve development is not observed in the most distal 6 pre-vertebrae.


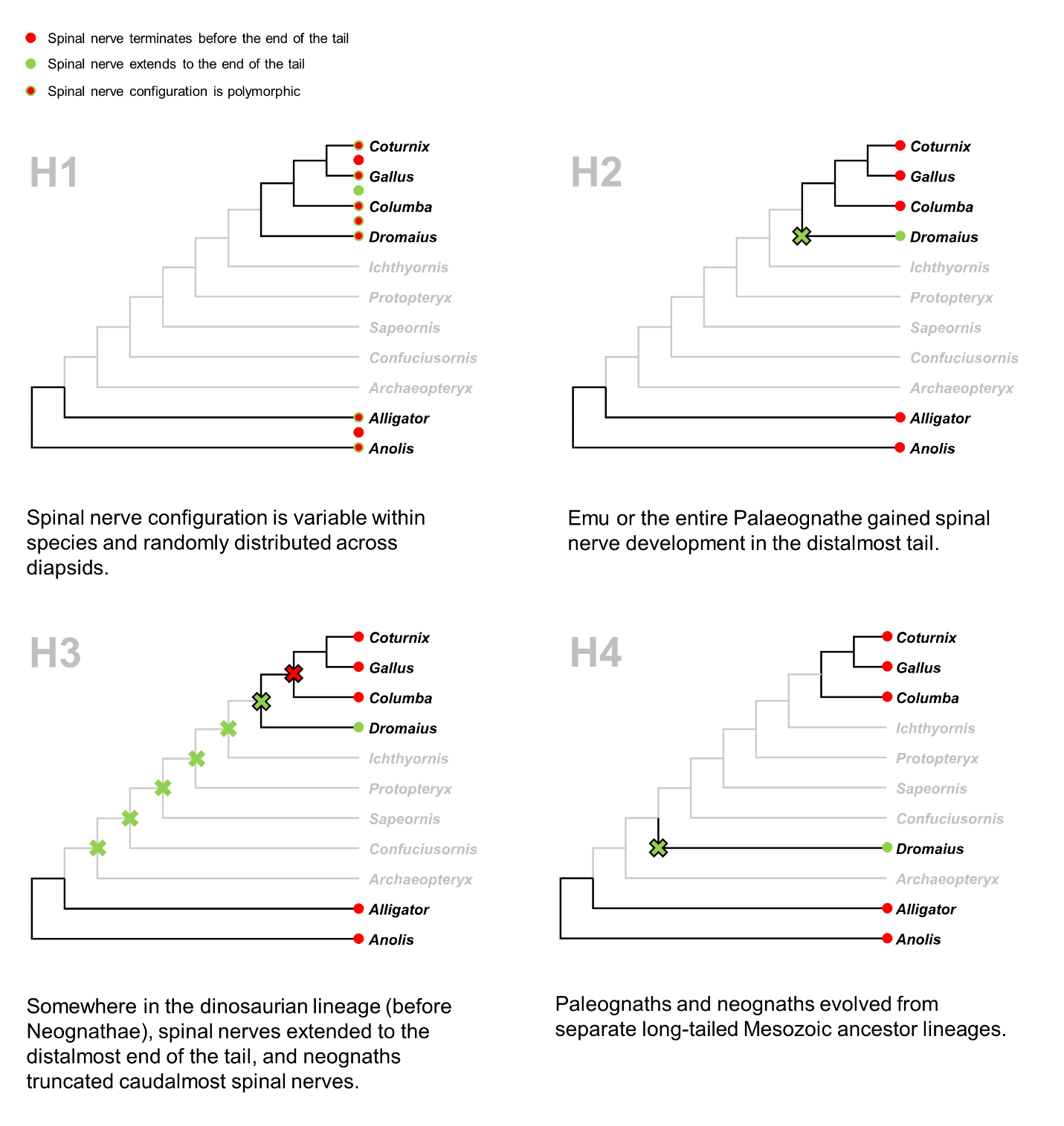


**Supplementary Figure 3. Phylogenetic representation of evolutionary hypotheses for avian spinal nerve configuration.** (cladograms modified from Wang and Lloyd, 2016)

**H1.** This is effectively the null hypothesis, with random distribution of spinal nerve configuration, and our sample size is too small to reveal this situation. Variable spinal nerve configuration could occur by either multiple, independent gains (or losses) of distalmost spinal nerve generation, or by the same mutation(s) causing alternative nervous system development in similar but not identical genetic backgrounds (as seen with the same mutation causing different phenotypes in different mouse species). While H1 is possible, the combined spinal nerve data and the data indicating that neognaths possess fully fused pygostyles but paleognath pygostyles (at least those examined) have spinal cord-aligned foramina makes this hypothesis less likely.

**H2.** The hypothesis that mutations in emus or paleognaths as a group caused extended spinal nerve development is impacted by two opposing factors: 1) it is far more likely for a mutation to terminate a process than to build a complicated, highly orchestrated process such as axial extension, and 2) the tail is a region that can sustain more mutations without significantly affecting viability. Many possibilities are inherent with this hypothesis, including a tail lengthening in Paleognathae followed by another tail shortening event. Our data is consistent with this overall hypothesis.

**H3.** This hypothesis is predicated on a shared long-tailed ancestor, but at some point before Neognathae the spinal nerve configuration was like emu, with spinal nerve development to the distalmost end of the tail. Neognathous birds would then have incurred additional mutation(s) to truncate caudal spinal nerves. Several possibilities are included in this hypothesis, such as a long-tailed ancestor with extended caudal spinal nerves (less possible considering our alligator and anole data), or a tail shortening event resulting in extended spinal nerves from a long-tailed ancestor with truncated spinal nerves.

**H4.** The concept of separate long-tailed Mesozoic ancestors of modern birds (at least one for neognaths and at least one for paleognaths) is consistent with our collective data, but includes multiple convergent pygostyle-forming events in avian evolution.

**Most likely hypothesis?** The degree of complication of these scenarios, including the fact that from a developmental perspective, the different proposed evolutionary events are not equal in probability, means that likelihood is difficult to assess. That being said, H2 and H4 are probably equally parsimonious, everything considered, but evolution often takes a more circuitous route than the simplest explanation. More avian fossils need to be discovered and analyzed, and more morphological traits need to be examined for possible convergence using an evo-devo approach between neognaths and paleognaths to reach any justifiable conclusions at this point.

Wang M, Lloyd GT (2016) Rates of morphological evolution are heterogeneous in Early Cretaceous birds. *Proc Biol Sci,* **283**.
